# Single-Cell Profiling of Latently SIV-Infected CD4^+^ T Cells Directly *Ex Vivo* to Reveal Host Factors Supporting Reservoir Persistence

**DOI:** 10.1128/spectrum.00604-22

**Published:** 2022-05-05

**Authors:** Andrey Tokarev, Kawthar Machmach, Matthew Creegan, Dohoon Kim, Michael A. Eller, Diane L. Bolton

**Affiliations:** a US Military HIV Research Program, Walter Reed Army Institute of Research, Silver Spring, Maryland, USA; b The Henry M. Jackson Foundation for the Advancement of Military Medicine, Bethesda, Maryland, USA; Karolinska Institutet

**Keywords:** host-pathogen interactions, latent infection, reservoir, simian immunodeficiency virus, single-cell

## Abstract

HIV-1 cure strategies aiming to eliminate persistent infected cell reservoirs are hampered by a poor understanding of cells harboring viral DNA *in vivo*. We describe a novel method to identify, enumerate, and characterize in detail individual cells infected *in vivo* using a combination of single-cell multiplexed assays for integrated proviral DNA, quantitative viral and host gene expression, and quantitative surface protein expression without any *in vitro* manipulation. Latently infected CD4^+^ T cells, defined as harboring integrated provirus in the absence of spliced viral mRNA, were identified from macaque lymph nodes during acute, chronic, and combination antiretroviral therapy (cART)-suppressed simian immunodeficiency virus (SIV) infection. Latently infected CD4^+^ T cells were most abundant during acute SIV (~8% of memory CD4^+^ T cells) and persisted in chronic and cART-suppressed infection. Productively infected cells actively transcribing viral mRNA, by contrast, were much more labile and declined substantially between acute and chronic or cART-suppressed infection. Expression of most surface proteins and host genes was similar between latently infected cells and uninfected cells. Elevated *FLIP* mRNA and surface CD3 expression among latently infected cells suggest increased survival potential and capacity to respond to T cell receptor stimulation. These findings point to a large pool of latently infected CD4^+^ T cells established very early in acute infection and upregulated host factors that may facilitate their persistence *in vivo*, both of which pose potential challenges to eliminating HIV-1 reservoirs.

**IMPORTANCE** Effective combination antiretroviral therapy controls HIV-1 infection but fails to eliminate latent viral reservoirs that give rise to viremia upon treatment interruption. Strategies to eradicate latently infected cells require a better understanding of their biology and distinguishing features to promote their elimination. Tools for studying these cells from patients are currently limited. Here, we developed a single-cell method to identify cells latently infected *in vivo* and to characterize these cells for expression of surface proteins and host genes without *in vitro* manipulation, capturing their *in vivo* state from SIV-infected macaques. Host factors involved in cell survival and proliferation were upregulated in latently infected cells, which were abundant in the earliest stages of acute infection. These studies provide insight into the basic biology of latently infected cells as well as potential mechanisms underlying the persistence of HIV-1/SIV reservoirs to inform development of novel HIV-1 cure strategies.

## INTRODUCTION

Effective combination antiretroviral therapy (cART) suppresses HIV-1 viremia but fails to cure patients of HIV-1 infection. Latently infected resting memory CD4 T cells are widely believed to be the primary barrier to eradicating the virus, and multiple mechanisms of persistence of these cells have been described (1, 2). Latent infection, defined here as cells with an integrated provirus but lacking evidence of viral gene expression to support replication, is established very early in primary infection and persists in infected individuals for life. These cells are not eliminated by cART, and virus production is reactivated typically within weeks after therapy discontinuation (3–8). Proposed strategies for attacking these viral reservoirs include shock and kill to reactivate and eliminate latently infected cells, block and lock to establish a state of deep latency, and endonuclease excision of integrated provirus to clear latently infected cells of provirus (9–12). However, implementation of such approaches often requires biomarkers that distinguish latently infected cells to enable specific targeting of the intervention, and definition of these biomarkers is lacking. In addition, detailed molecular and phenotypic characterization of cells infected *in vivo* may elucidate mechanisms of viral persistence and aid in the development of novel therapies.

Several challenges limit studies of latently infected cells infected *in vivo*. First, under suppressive therapy, the frequency of these cells is estimated to be very low, ranging from 10^−3^ to 10^−6^ in resting memory CD4 T cells, as determined by quantification of integrated proviral DNA quantitative PCR (qPCR) and quantitative viral outgrowth assay (QVOA), respectively (13, 14). Second, no known surface or cytoplasmic markers uniquely identify latently infected cells, preventing high-throughput isolation. Third, defining what constitutes latent cellular infection is complicated by evidence of multiple blocks to viral transcription, translation, or virion production (15). Such a spectrum of potential states of latency suggests that a combination of viral RNA/DNA as well as viral protein-based assays may be required to definitively identify latently infected cells.

Despite these challenges, several biomarkers of latently infected memory CD4 T cells have been identified using multiple approaches (16, 17). General characteristics described to date include increased expression of type I interferon (IFN)-regulated genes (18), increased resting state (19), and memory subsets with high survival or proliferation potential, although naive cells may also be infected (20–23). Specific T helper functional subsets, Th17, Th1/Th17, and T follicular helper cells, as defined by CCR6, CXCR3 plus CCR6, and CXCR5 plus PD-1 expression, respectively, also appear enriched for latently infected cells (24–28). Other markers include immune checkpoint proteins, PD-1, LAG-3, CTLA-4, and TIGIT, interferon-induced transmembrane protein 1 (IFITM1), adhesion and costimulatory molecule CD2, and low-affinity immunoglobulin gamma Fc region receptor CD32a (29–32). While these findings suggest that latently infected cells tend to exhibit certain physical and immunologic properties, they also indicate a heterogeneous phenotype characterized by multiple possible markers.

The methods used to date to profile infected cells vary and generally fall into three categories: (i) *in vitro* infection models to generate latently infected cells for characterization (33–37), (ii) *ex vivo* purification of cells expressing a specific marker hypothesized to enrich for infected cells (e.g., memory cells) followed by viral quantitation in the target population (20, 24–26, 38, 39), and (iii) *in vitro* reactivation of latently infected cells, followed by detection of viral RNA or protein expression for detailed characterization by flow cytometry or gene expression (40–43). There are limitations to these approaches. First, it is unclear how *in vitro* latent infection models or *ex vivo* reactivation of latent infection recapitulate the properties of cells infected *in vivo* (33, 44, 45). Bulk cell analyses are hampered by averaging signals across heterogeneous mixtures of uninfected and infected cells, preventing definitive identification of individual cell features. *In situ* hybridization strategies combining RNA- and DNA-scope are able to identify latently infected cells (46) but tend to be limited with respect to the number of host cell parameters assessed.

To identify and phenotype latently infected cells directly *ex vivo* without any *in vitro* manipulation, we developed a single-cell method that harvests both host cell DNA and RNA and combines this information with host cell gene and surface protein expression. Building on our previously described targeted single-cell protein and transcript evaluation (tSCEPTRE) method (47, 48), single cells are sorted by multiparametric flow cytometry, capturing protein expression, followed by qPCR detection of integrated proviral DNA (ipDNA) and highly multiplexed reverse transcriptase quantitative PCR (RT-qPCR) detection of viral and host RNA. Latently infected cells, defined as cells containing ipDNA and unspliced viral RNA below levels seen in productive infection, and lacking multiply spliced viral RNA (*tat/rev*), were identified in macaque lymph nodes spanning acute, chronic, and cART-suppressed simian immunodeficiency virus (SIV) infection. The frequency of latently infected memory CD4 T cells was greatest in acute infection and lowest following cART suppression. Unlike replication-active cells, latently infected cells were double-positive for surface CD3 and CD4 and lacked upregulation of activation markers. Analysis of host gene mRNA levels revealed increased expression of the prosurvival factor *FLIP* in latently infected cells in both acute and chronic infection. Overall, latently infected and uninfected memory CD4 T cells exhibited similar profiles, though differences noted in both host gene and protein expression demonstrate the power and sensitivity of this approach to distinguish novel features of infected cells directly *ex vivo* and elucidate potential mechanisms of their persistence.

## RESULTS

### Sensitivity and specificity of assay to identify latently infected single cells.

**FIG 1:**
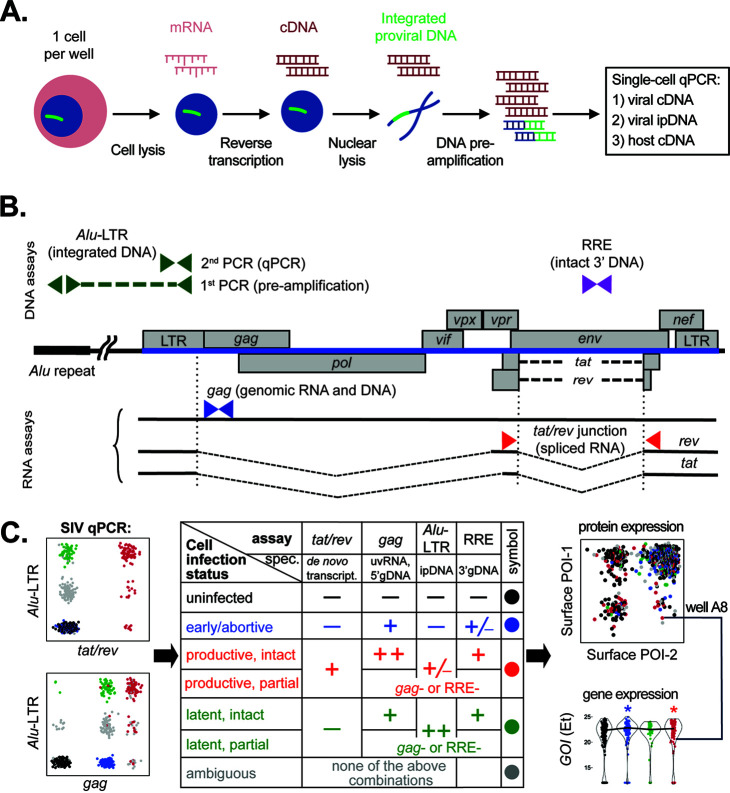
Multiplexed RNA and ipDNA qPCR approach to identify SIV-infected cells across multiple viral life cycle stages. (A) Schematic of the method workflow. Single memory CD4 T cells from cryopreserved specimens are sorted by flow cytometry into 96-well PCR plates containing reverse transcription-preamplification reaction components and gene-specific primers (host and viral). RNA reverse transcription (RT), brief cDNA preamplification (3 cycles), nuclear lysis via subsequent proteinase K addition, and further preamplification (15 cycles) are followed by qPCR. (B) qPCR assays used to classify cellular infection stages. SIV genome integrated in rhesus macaque DNA is shown with individual genes relative to the nearest *Alu* repeat. Assays for viral RNA, *tat*/*rev* and *gag*, and primer positions are shown below. Assays for viral DNA are shown above. For *Alu*-LTR assay, green dashed line indicates integration-specific amplification during preamplification stage. Green arrowheads above indicate primer positions for the second PCR-qPCR, not specific for ipDNA. (C) Infected cell classification scheme. The preamplified cDNA and ipDNA mixture is analyzed by qPCR for the presence of viral nucleic acids: *Alu*-LTR versus *tat/rev* (top), *Alu*-LTR versus *gag* (bottom), and others as shown in panel B. Cells are classified into the indicated infection status by the presence and relative amount of viral nucleic acids, as indicated in the table in the middle of the panel. Gene expression data (bottom, right; GOI, gene of interest) are combined with FACS data (top, right; POI, protein of interest) obtained during cell sorting indexed by well location. spec., specificity; uvRNA, unspliced viral RNA; gDNA, genomic DNA; ipDNA, integrated proviral DNA.

To develop a method able to identify and characterize individual latently infected cells directly *ex vivo*, we modified our tSCEPTRE approach by incorporating a nuclear lysis step and adding qPCR detection of integrated provirus (Fig. 1A) (47–49). As in tSCEPTRE, cells were sorted by flow cytometry at one cell per well into a reaction mix for cell lysis, RNA reverse transcription using gene-specific primers including viral mRNA (e.g., *gag* and *tat/rev*), and cDNA preamplification followed by quantification by qPCR (48) (Fig. 1B). To harvest ipDNA, the nuclear envelope was lysed by proteinase K following three rounds of cDNA preamplification, and *Alu-*long terminal repeat (LTR) primers were then used to preamplify ipDNA during the remaining cDNA preamplification cycles. The infection status of each cell was determined by the combination of viral genes present in the resulting cDNA and genomic DNA (Fig. 1C). We hypothesized that latently infected cells would be distinguished by the presence of ipDNA and the absence of viral gene expression (e.g., multiply spliced *tat/rev* mRNA).

**FIG 2:**
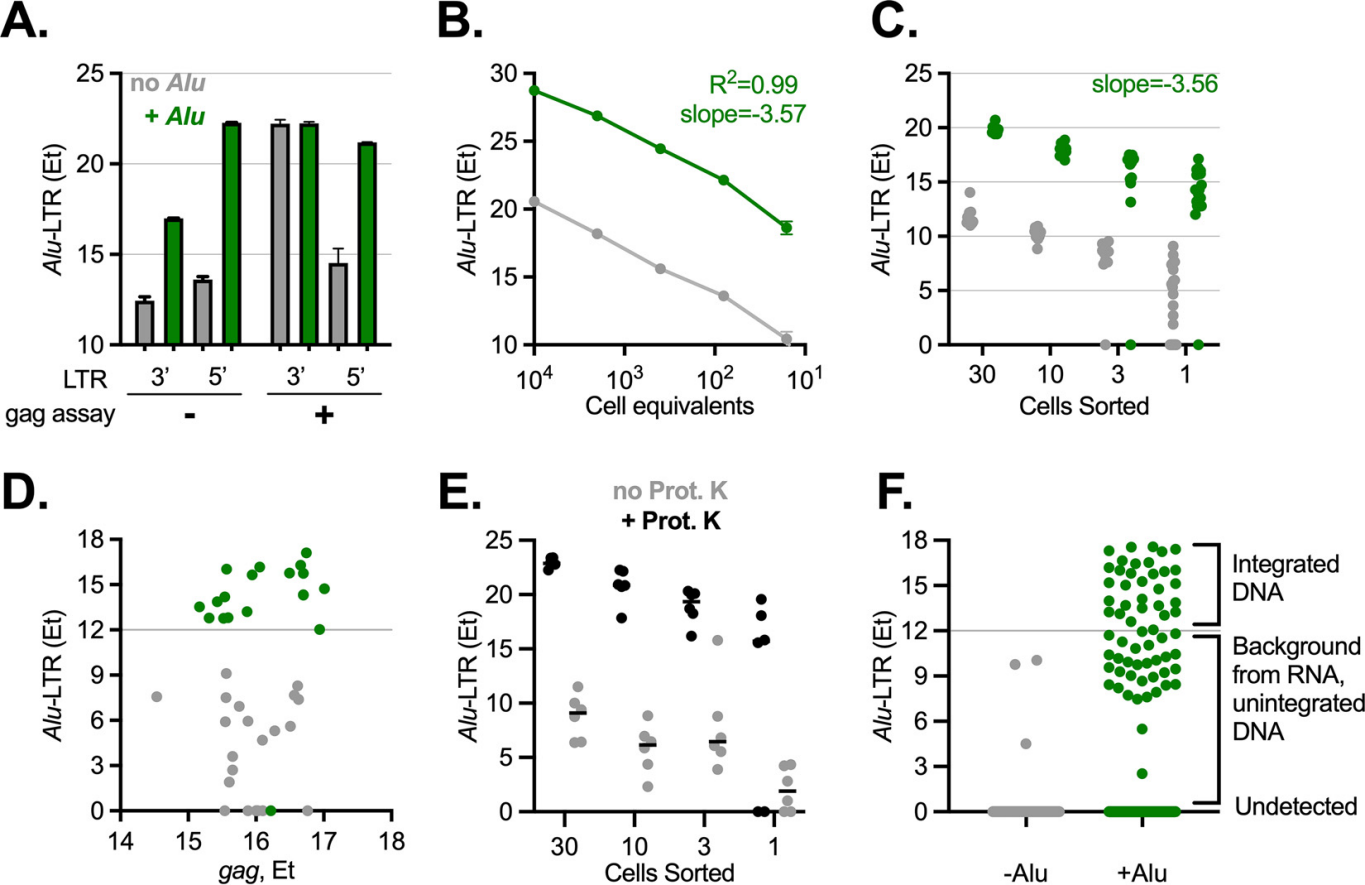
Validation of integrated SIV DNA PCR assay with single-cell sensitivity. (A) Comparison of *Alu*-LTR assays targeting *Alu* sequences 5′ and 3′ of integrated provirus. 3D8 cell DNA was extracted and 30 cell equivalents were used as the template in the protocol described in Fig. 1, including (green) or omitting (gray) *Alu* primers with or without the *gag* assay. LTR primers annealing to the same sequences within 5′ or 3′ LTR, both extending outward of the viral DNA, were compared. (B) Qualification of *Alu*-LTR using DNA dilution series. DNA from cell line 3D8 was extracted, serially diluted, and used in protocol described in Fig. 1, including or omitting *Alu* primers. Cell number equivalents to template DNA mass are plotted on the *x* axis, assuming 100% efficient cell and nuclear lysis. Linear regression slope and R^2^ value for “+*Alu*” condition are indicated. (C) Qualification of *Alu*-LTR using sorted cells. 3D8 cells sorted by flow cytometry directly into reaction mix at the indicated cell dilutions were assessed for integrated SIV DNA by *Alu*-LTR qPCR as described in Fig. 1 in the presence or absence of *Alu* primers. (D) Sensitivity and specificity of the *Alu*-LTR assay at the single-cell level. *Alu*-LTR *E_T_* values obtained for single 3D8 cells described in panel C are plotted against *gag* *E_T_* values. Horizontal line at *E_T_* = 12 demarcates threshold for *Alu* primer-dependent signal, indicating ipDNA. (E) *Alu*-LTR signal achieved with and without nuclear lysis. Live 3D8 cells were processed as in panel C, including or omitting nuclear lysis with proteinase K. (F) Specificity of *Alu*-LTR assay in primary CD4 T cells latently infected *in vivo*. Single cells from a chronically SIV-infected rhesus macaque lymph node were processed as described in Fig. 1 and in the presence (*n* = 592) or absence (*n* = 176) of *Alu* primers. Annotation at right indicates integrated DNA classification status applied to macaque samples.

Since SIV infection in nonhuman primate models recapitulates many aspects of latent HIV-1 infection in humans (50–53), including host cell tropism, viral persistence during cART, and much of the viral life cycle, we developed our approach for the study of latent SIV infection given the large number of intact proviral genomes in this model. Candidate qPCR assays specific for integrated SIV provirus were designed based on a previously described HIV-1 *Alu*-LTR assay widely used for quantification of ipDNA in bulk cells (38, 39). To multiplex this assay with other viral assays and minimize potential primer interactions between assays, *Alu*-LTR was selected over *Alu*-*gag* (38, 54, 55), as the *gag* region was used for detecting 5′ DNA and unspliced viral RNA. A downselected *Alu-*LTR assay was validated to ensure signal specificity, linear amplification, sensitivity at a single-cell level, and lack of interference with other viral (RT-)qPCR assays (Fig. 2). First, amplification of the 5′ LTR was chosen over the 3′ LTR based on comparison of *Alu*-LTR qPCR signal obtained with LTR primers oriented 5′ or 3′ of the provirus, respectively, and in the presence and absence of *Alu* and *gag* primers, using the 3D8 cell line containing one copy of integrated SIVmac316 as the template (Fig. 2A) (55). Both the 5′ and 3′ LTR assays exhibited robust increases with *Alu* priming when the *gag* assay was excluded, demonstrating *Alu*-dependent amplification. Low-level background *Alu*-LTR qPCR signal was observed in the absence of *Alu* primer, as expected, due to unidirectional DNA synthesis from the LTR primer (38, 39, 54). When the *gag* primers were included to simulate the multiplexed RT-qPCR conditions, *Alu-*specific priming was maintained for the 5′ LTR assay but not the 3′ LTR assay (likely due to production of LTR-*gag* amplicons). The *Alu-*specific 5′ LTR ipDNA signal was ~256-fold higher (i.e., ~8 *E_T_*, where *E_T_* = 40 − *Ct* and *Ct* is the cycle threshold; *E_T_*, expression threshold) than the background observed with the 5′ LTR primer alone.

To validate the assay for use in single-cell applications, we measured performance on limiting 3D8 cell template. Using serial dilutions of bulk 3D8 cell DNA, *Alu*-LTR amplification efficiency was high (90.5%) and reproducible (Fig. 2B, slope −3.57), but sensitivity below 10 cells was poor using extracted DNA template. To improve recovery at low cell numbers, 3D8 cells were sorted by flow cytometry at dilutions of 1 to 30 cells per well and processed as shown in Fig. 1A. Again, ipDNA qPCR exhibited linear amplification (Fig. 2C, slope −3.56). Background in the absence of Alu primer was again; 8 *E_T_* lower. Sensitivity within single cells was robust: integrated provirus was detected in 18 of 19 (95%) replicate single cells, as identified by SIV *gag* positivity, which served as a control for efficient nucleic acid recovery (Fig. 2D). Nuclear lysis was required for *Alu*-LTR amplification as signal approached background (no *Alu*) in the absence of proteinase K (Fig. 2E). Median *E_T_* difference between conditions with and without *Alu* primers was 10 (2^10^ copies). ipDNA *E_T_* variability increased with cell dilution, likely due to stochastic detection at low-copy conditions, and spanned ~5 *Ct* for 1-cell samples. The lowest positive single-cell *Alu*-LTR *E_T_* value was 12, suggesting that an *E_T_* value of ≥12 serves as a threshold for identifying provirus-positive cells. We validated this threshold using memory CD4 T cells from a chronically SIV-infected rhesus macaque lymph node. Background *E_T_* values did not exceed 10 in the absence of *Alu* primers, supporting an *Alu*-LTR *E_T_* value of ≥12 as an appropriate cutoff in *ex vivo* specimens (Fig. 2F). Taken together, these data indicate that the method is both sensitive and specific for SIV-infected cells harboring ipDNA and therefore appropriate for quantitative and qualitative assessments.

### Detection of latent SIV-infected rhesus cells during acute, chronic, and cART-suppressed infection.

**FIG 3:**
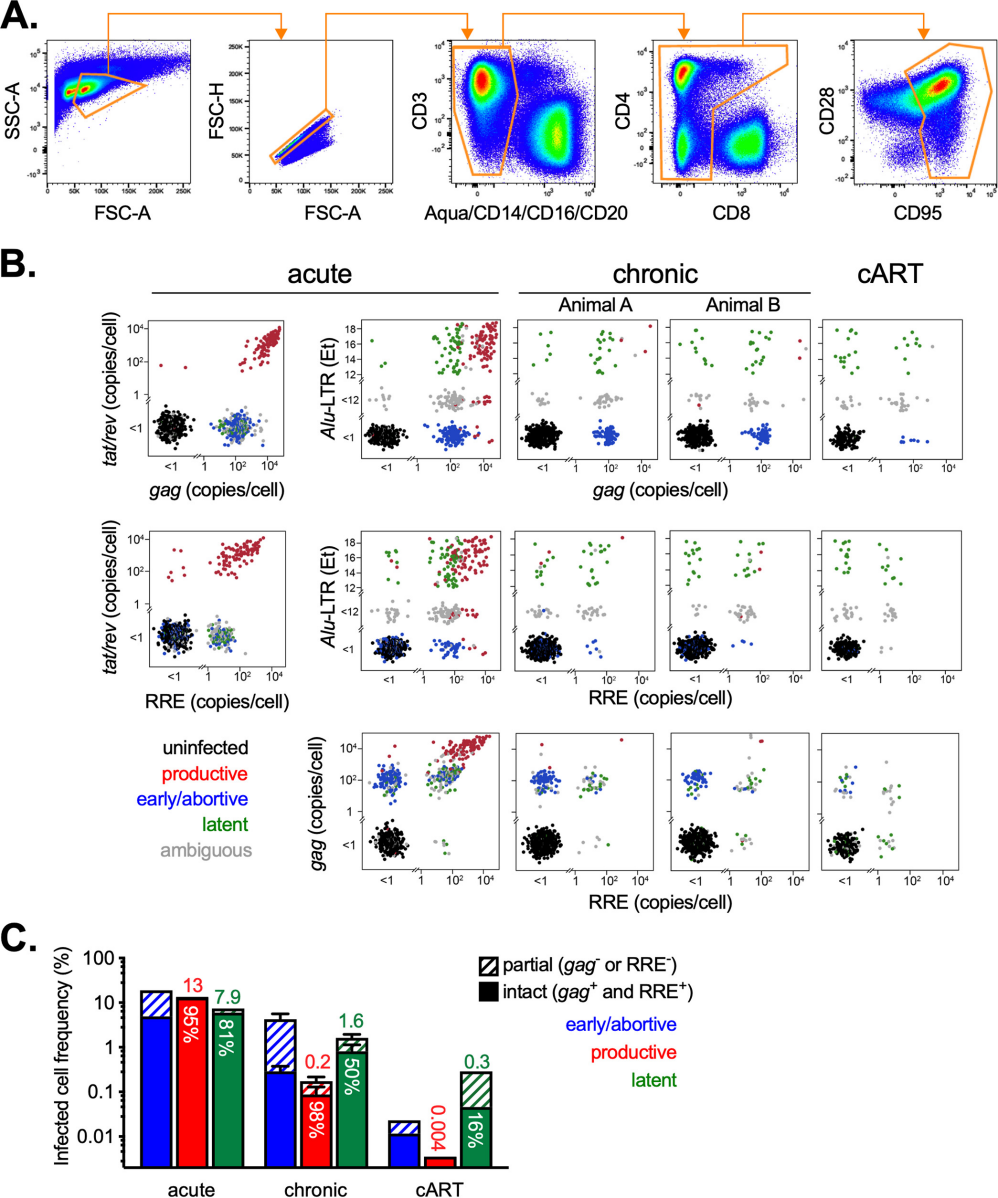
Isolation, identification, and quantification of latently infected macaque CD4 T cells harboring SIV DNA directly *ex vivo*. (A) Gating tree used to sort single memory CD4 T cell (CD8-negative) T lymphocytes from macaque lymph nodes. (B) Identification of latently infected cells from acute (*n* = 1), chronic (*n* = 2), and cART-treated (*n* = 1) SIV infection of macaques by viral gene qPCR. Lymph node mononuclear cells were processed as in Fig. 1A using single-cell flow cytometric sorting. For cART-treated infection, replicate limiting dilution cell sorting was used to select wells containing a single cell positive for *gag* or *Alu*-LTR by probability statistics (cell dilution with <33% positivity rate for *gag* or *Alu*-LTR). Single-cell *tat/rev*, *gag*, and RRE copies are shown in bivariate plots against *Alu*-LTR *E_T_* values for each specimen. *Alu*-LTR *E_T_* of <12 background values and undetected viral gene qPCR signal were binned and randomly scattered for visualization. (C) Frequency of infected memory CD4 T cells in macaque lymph nodes shown in panel B classified by cellular infection status. Bar shading indicates the fraction of each infected cell group harboring intact (solid) versus partial (striped) genomes. The frequency of latent and productive T cell infection is indicated above each bar; the percentage of latently infected cells with intact proviral genomes is indicated in white text within the green bars.

To study latently infected cells directly *ex vivo* with single-cell resolution using this method, memory CD4 T cells were isolated by fluorescence-activated cell sorting (FACS) from four SIV-infected rhesus macaque lymph node specimens. Since SIV downregulates both CD3 and CD4 from the surface of infected T cells (56, 57), cells lacking CD3 and CD4 were included in the gating strategy (Fig. 3A). To distinguish latently infected cells from productively infected cells, both of which contain ipDNA, the initial RT-preamplification reaction included primers for spliced (*tat/rev*) and unspliced (*gag*) viral RNA for downstream quantitation by qPCR. Transcriptionally silent latently infected cells are expected to be negative for *tat/rev* and low for *gag*. In addition, to assess provirus “intactness,” the preamplified DNA was evaluated by a Rev response element (RRE)-specific qPCR assay targeting the 3′ half of the genome (Fig. 1B), where large deletions are the most commonly occurring proviral mutation (58).

Given the robust viral replication and relatively abundant cellular infection during acute infection, we analyzed a specimen collected at approximately peak viremia to facilitate identification of infected cells, including differentiation of latently infected cells from transcriptionally active cells, which we refer to as productively infected. Memory CD4 T cells (*n* = 768) from a mesenteric lymph node biopsy specimen of an SIVmac251-infected macaque (day 10 postinfection) were sorted and processed as described in Fig. 1A. Single-cell qPCR for the four viral gene assays (*tat/rev*, *gag*, RRE, *Alu*-LTR) revealed distinct infected cell populations, defined by the quantitative signals from each assay (Fig. 3B, “acute”). These clusters were interpreted to represent cells in discrete infection stages as follows: (i) productive or replication-active infection, as defined by *tat/rev* expression, which generally coincided with both high unspliced genomic RNA content (*gag* copies per cell > ~10^3^) and robust *Alu*-LTR signal, (ii) early, abortive, or defective provirus infection, as defined by undetected *Alu*-LTR and *tat/rev*, but intermediate levels of genomic RNA (0 < *gag* copies per cell < ~10^3^), (iii) latent infection, as defined by an *Alu-*LTR *E_T_* value of >12, lack of *tat/rev*, and low or intermediate genomic RNA (*gag* copies per cell < ~10^3^), and (iv) uninfected, as defined by negative qPCR for all SIV assays. Cells that did not satisfy any of the above criteria were deemed ambiguous and excluded from further analysis. Among productively and latently infected cells, positivity for both *gag* and RRE, representing 5′ and 3′ DNA, respectively, was used to define intact provirus.

Using these criteria, replication-active SIV-infected cells represented 13% of memory CD4 T cells (*n* = 103) in this lymph node specimen, consistent with prior estimation using an independent limiting dilution method (47, 48) (Fig. 3C). Latently infected cells comprised 8% (*n* = 61), while early/abortive/defective infection was most abundant at 18% (*n* = 142). Thus, in total, ~40% of memory CD4^+^ T cells were infected in this secondary lymphoid tissue during early acute infection. The majority of productively and latently infected cells were also positive for RRE and *gag*, indicating intact 5′ and 3′ regions of the provirus (Fig. 3C).

To further confirm the presence of integrated provirus in cells identified as productively or latently infected, we sequenced the integration site *Alu-*LTR PCR amplicon for ~30 single cells. Sequencing reads aligning to both Macaca mulatta and SIVmac251 spanning the presumed integration site were obtained for 5 cells, all with relatively high *Alu*-LTR *E_T_* values (Table 1; poor quality sequence data limited integration site identification for the other 25 cells). Three of these cells contained one integration site each, while two integration sites were identified per cell for two cells (one productive and one latent). Integration sites were identified in introns of host genes *ARHGAP45*, *RBM33*, *MOB3A*, and *AKTIP* and in intergenic regions, consistent with SIV and HIV-1 integration sites located both inside and outside genes (59). Of note, *RBM33* was previously identified as an integration hot spot for HIV-1 (60). These findings support the presence of integrated provirus in cells identified by our single-cell *Alu-*LTR qPCR method.

**TABLE 1:** Integration site sequencing analysis of single SIV-infected cells^*a*^

Cell ID	*Alu*-LTR *E_T_*	*gag* *E_T_*	Infection status	Distance between *Alu* and LTR (nt)	Homology with rhesus DNA (nt)	Gene, position within gene
4G6	18.6	27.0	Productive	146	146	*ARHGAP45*, intron
4G6	18.6	27.0	Productive	147	147	*RBM33*, intron
5H8	18.5	22.1	Productive	173	170	*MOB3A,* intron
1C6	16.9	16.6	Latent	18	18	ND
6B5	17.8	21.3	Latent	143	143	intergenic
6B5	17.8	21.3	Latent	183	183	intergenic
2A6	17.2	19.4	Latent	133	106	*AKTIP*, intron

a Host gene integration sites were determined by Sanger sequencing of five memory CD4 T cells isolated from a rhesus macaque lymph node collected 10 days post-SIVmac251 infection. nt, nucleotides; ND, not determined.

We next examined cells from chronic infection when the dynamics between latent and productive cellular infection are expected to differ from the acute stage due to more limited virus replication. Lymph node memory CD4 T cells from two chronically SIV-infected macaques (7 months postinfection) were sorted and evaluated as described above (*n* = 1,728 cells). Overall, fewer infected cells were observed during chronic infection than during acute infection. Productively infected cells were less abundant in both animals in chronic infection, representing ~0.2% of memory CD4 T cells (Fig. 3B and C), over a log reduction compared to those observed in acute infection. Latently infected cells outnumbered productively infected cells by ~8-fold, at 1.6% in both animals. Early/abortive infected cells remained the most abundant population of infected cells, averaging 4.3% of memory CD4 T cells. Approximately half of the latently infected cells contained intact 5′ and 3′ genomes.

Lastly, we measured infected cells during suppressive cART, which is expected to further limit viral replication and productive cellular infection. Lymph node memory CD4 T cells were analyzed in an animal infected with SIVmac251 that initiated cART 8 weeks postinfection, with suppressed viremia for 5 weeks at the time of biopsy (15 weeks postinfection). Due to the much lower frequency of infected cells in this setting relative to that in acute and untreated chronic infection (61), we identified cell dilutions containing one infected cell per well by replicate limiting dilution cell sorting and Poisson distribution probability estimation. These multicell wells were used for downstream analysis in lieu of single-cell wells for this specimen. For example, <33% of replicate 100-cell wells were positive for any viral gene assay; these wells were therefore deemed unlikely to contain more than one infected cell and represent single infected cells for the purpose of viral gene profiling. As expected, productively SIV-infected cells were extremely rare, estimated at 0.004% of memory CD4 T cells (Fig. 3C). Latently infected cells were more abundant, representing 0.3%. The proportion of provirus-intact latently infected cells (RRE^+^ and *gag*^+^) was lower in the cART setting than in acute and untreated chronic infection, comprising only 16% of all latently infected cells, indicating greater accumulation of proviral deletions in suppressed infection (53). Early/abortive infection was ~10-fold less common than latent infection (0.02%), consistent with cART blockade of new rounds of cellular infection.

### Flow cytometric surface protein profiling of latently infected single cells.

**FIG 4:**
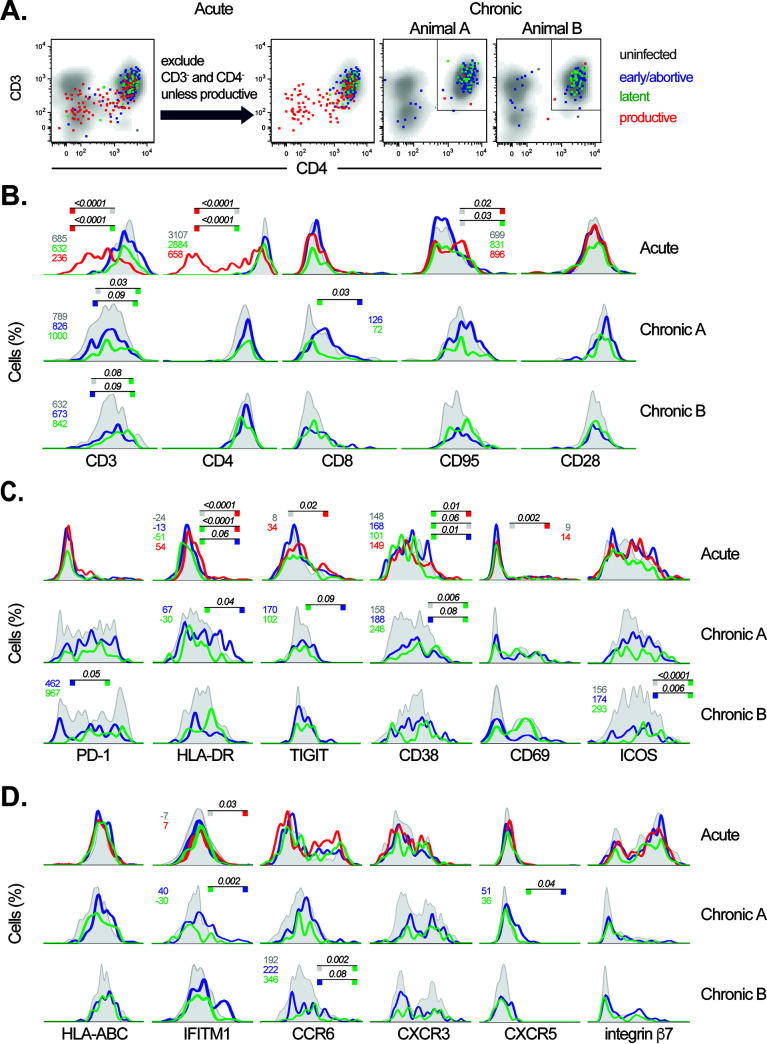
Surface protein expression profile of latently SIV-infected CD4 T cells. (A) Surface expression of CD4 and CD3 assessed by flow cytometry is shown for memory CD4 T cells isolated from lymph nodes of SIV-infected macaques. SIV-infected cell classification is indicated by dot color (SIV^+^ cells); gray density shading depicts uninfected cells. Cell selection process for surface protein profiling analysis is shown for acute (left; *n* = 1) and chronic (right; *n* = 2) infection specimens. Rectangular gate depicts CD3^+^, CD4^+^ double-positive CD4 T cells included in analysis of cells from chronic infection. (B to D) Surface protein expression profiles are shown as histograms for each infected cell group, indicated by line color, and overlaid on that of uninfected cells (gray). Productively infected cells are not shown for the chronic infection specimens due to small population size. Significant differences between the latently infected cells and the other populations (all three animals) as well as between productively infected and uninfected cells (acute infection animal only) are indicated (*P_adj_* < 0.1, Dunn test with Benjamini-Hochberg false-discovery rate [FDR] adjustment). Cell population comparisons are indicated by colored blocks at line termini. Median fluorescent intensity values of relevant cell populations are reported in corresponding colored text.

To identify host proteins differentially expressed on the surface of infected CD4 T cells, we merged the viral RNA/DNA PCR data with the protein expression data collected by FACS for each cell from all untreated animals. It was not possible to apply this approach to the cART-suppressed animal due to the presence of uninfected cells combined with each infected cell in the bulk cell collection. Cells were grouped by infection status as described above. We first examined CD3 and CD4 expression, as cells lacking these proteins potentially introduce non-CD4^+^ T cell contaminants in the analysis. While most infected cells were double-positive for CD3 and CD4, many *tat/rev^+^* cells expressed low levels of these surface proteins (Fig. 4A). Given the well-established downmodulation of both CD3 and CD4 by SIV (56, 62, 63), we attributed the reduced expression of these markers on *tat/rev*^+^ cells to viral protein expression rather than infection of cells other than CD4^+^ T cells. Therefore, for comparison purposes, we determined CD3, CD4 double-positive cells to be the appropriate uninfected reference population and excluded cells negative for either CD3 or CD4 and *tat/rev* from the uninfected cell group from further analysis (Fig. 4A). For the purposes of differential protein and gene expression analysis, RRE detection was not used as a discriminating factor. This approach maximized statistical power through inclusion of all cells containing integrated provirus.

Expression of 17 surface proteins was compared among cells in each of the infection classifications during acute and chronic infection. Overall, these proteins, which included memory, activation, homing, and exhaustion markers, exhibited similar expression patterns between the uninfected and infected cell groups (Fig. 4B to D). However, several differences were noted in comparisons between uninfected cells and productively infected cells as well as between latently infected cells and the other cell populations. Relative to uninfected cells, productively infected cells expressed less surface CD3 and CD4, as noted above. They also expressed increased CD95 (47, 48), activation-associated proteins HLA-DR, TIGIT, CD38, and CD69, and a reported latency marker, interferon-induced transmembrane protein 1 (IFITM1) (30). These data are consistent with preferential targeting of activated T cells by SIV (64), though activation was not required for productive infection as many cells were negative or dim for activation markers.

Latently infected cells were double-positive for CD3 and CD4 in both acute and chronic infection with no evidence of downmodulation (Fig. 4A and B), consistent with limited viral protein expression in these cells and further supporting their latent classification. CD3 was expressed at higher levels on latently infected cells than on uninfected cells in the two chronically infected animals (median fluorescence intensity [MFI] of 1,000 versus 789 and 842 versus 632, adjusted *P* value [*P_adj_*] of 0.03 and 0.08, respectively, Table 2). Expression of CD3 on latently infected cells did not differ in the acute infection setting. CD38, ICOS, and CCR6 were also upregulated on the surface of latently infected cells during chronic infection. Relative to that on early/abortively infected cells, expression of CD3, PD-1, CD38, ICOS, and CCR6 was greater on latently infected cells in chronic infection, though only CD3 was consistently elevated in both animals. Latently infected cells expressed lower levels of CD8, HLA-DR, IFITM1, and CXCR5 than early/abortively infected cells in the chronic setting, and HLA-DR was also lower during acute infection. Other markers assessed, CD28, MHC class I, CXCR3, and integrin β7, did not exhibit differential expression. Taken together, in this univariate analysis, latently infected cells displayed an activation and homing profile overall similar to those of uninfected cells and productively and early/abortively infected cells, though expression of some activation markers varied.

**TABLE 2:** Median fluorescence intensity of surface protein staining on CD4 T cells from SIV-infected macaque lymph nodes^*a*^

Surface marker	Animal SIV infection status	Cell infection status (MFI)			
		Uninfected	Early/abortive	Latent	Productive
CD3	Acute	685	655	632	236
	Chronic animal A	789	826	1,000	NA
	Chronic animal B	632	673	842	NA
CD4	Acute	3,107	3,067	2,884	658
	Chronic animal A	3,277	3,787	3,474	NA
	Chronic animal B	2,740	2,886	2,547	NA
CD8	Acute	48	56	53	51
	Chronic animal A	91	126	72	NA
	Chronic animal B	60	93	76	NA
CD95	Acute	699	713	831	896
	Chronic animal A	803	1,047	836	NA
	Chronic animal B	677	742	898	NA
CD28	Acute	1,091	1,050	1,115	1,055
	Chronic animal A	1,408	1,544	1,358	NA
	Chronic animal B	857	945	958	NA
PD-1	Acute	14	10	16	20
	Chronic animal A	597	821	1,022	NA
	Chronic animal B	587	462	967	NA
HLA-DR	Acute	−24	−13	−51	54
	Chronic animal A	0	67	−30	NA
	Chronic animal B	31	18	80	NA
TIGIT	Acute	8	12	33	34
	Chronic animal A	98	170	102	NA
	Chronic animal B	156	131	220	NA
CD38	Acute	148	168	101	149
	Chronic animal A	158	188	246	NA
	Chronic animal B	186	238	289	NA
CD69	Acute	9	10	10	13.5
	Chronic animal A	103	164	140	NA
	Chronic animal B	54	21	97	NA
ICOS	Acute	−40	−4	−46	5
	Chronic animal A	40	87	138	NA
	Chronic animal B	156	174	293	NA
HLA-ABC	Acute	2,584	2,603	2,438	2,337
	Chronic animal A	1,809	2,386	2,084	NA
	Chronic animal B	1,022	1,196	1,059	NA
IFITM1	Acute	−7	−4	2	7
	Chronic animal A	−9	40	−30	NA
	Chronic animal B	12	36	18	NA
CCR6	Acute	172	172	228	229
	Chronic animal A	244	313	249	NA
	Chronic animal B	192	222	346	NA
CXCR3	Acute	46	37.5	65	46
	Chronic animal A	198	243	365	NA
	Chronic animal B	226	104	180	NA
CXCR5	Acute	38	39	35	42
	Chronic animal A	31	51	36	NA
	Chronic animal B	40	43	36	NA
Integrin b7	Acute	197	232	182	219
	Chronic animal A	46	62.5	52	NA
	Chronic animal B	28	75	34	NA

a Median fluorescence intensity (MFI) staining measured by flow cytometry is reported for each surface marker by animal and cell population. NA, not applicable.

**FIG 5:**
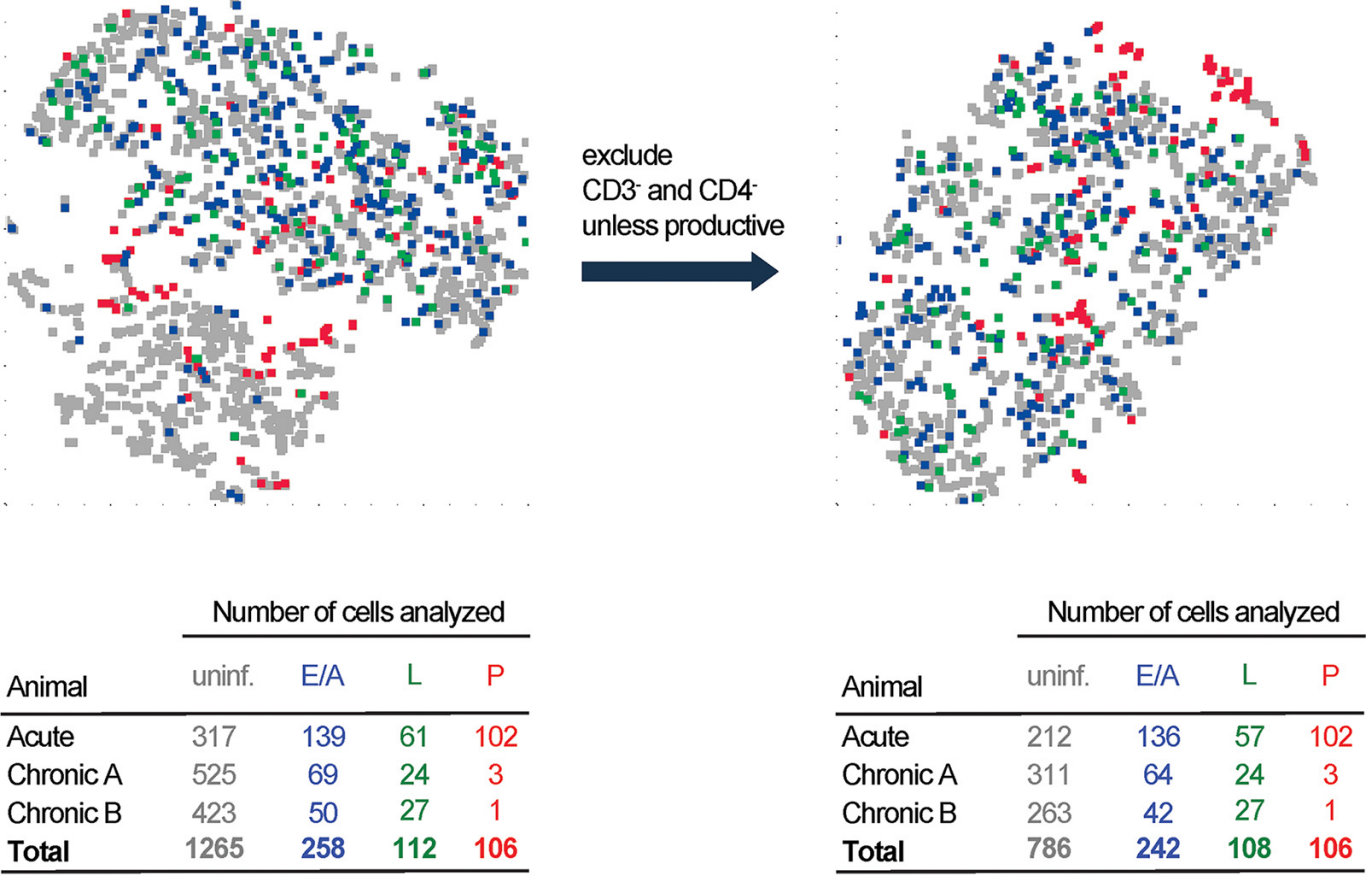
Composite surface protein expression profile of infected cells. *t-*distributed stochastic neighbor embedding (tSNE) plots representing clustering of the CD4 T cells across the different infection states from the three lymph node specimens described above. Analysis including (left) or excluding (right) CD3-negative and CD4-negative cells is shown. Number of cells in each infection group is indicated. Color coding is as in Fig. 4. Uninf., uninfected; E/A, early/abortive; L, latent; P, productive.

To identify potential combinations of surface proteins able to distinguish latently infected cells using a multivariate approach, we visualized the cells in two-dimensional space using *t*-distributed stochastic neighbor embedding (tSNE), a method for nonlinear dimensionality reduction. We analyzed a total of 1,265 uninfected, 258 early/abortively infected, 112 latently infected, and 106 productively infected cells combined from the three macaque lymph nodes sampled during acute and chronical infection described above. All 17 surface proteins specific for memory, activation, homing, and exhaustion markers were included in the analysis. Single-cell tSNE plots did not reveal infection status-specific clusters, as all of the infected cell groupings were heavily interspersed with one another and with uninfected cells (Fig. 5). The analysis was further refined by removing uninfected cells lacking CD3 and CD4, as described above in Fig. 4A. Uninfected cells and the infected cell groupings remained intermingled, providing additional evidence that phenotypic profiles for these markers are largely similar across these cellular infection states.

### Differential host gene expression among SIV-infected CD4^+^ T cells.

**FIG 6:**
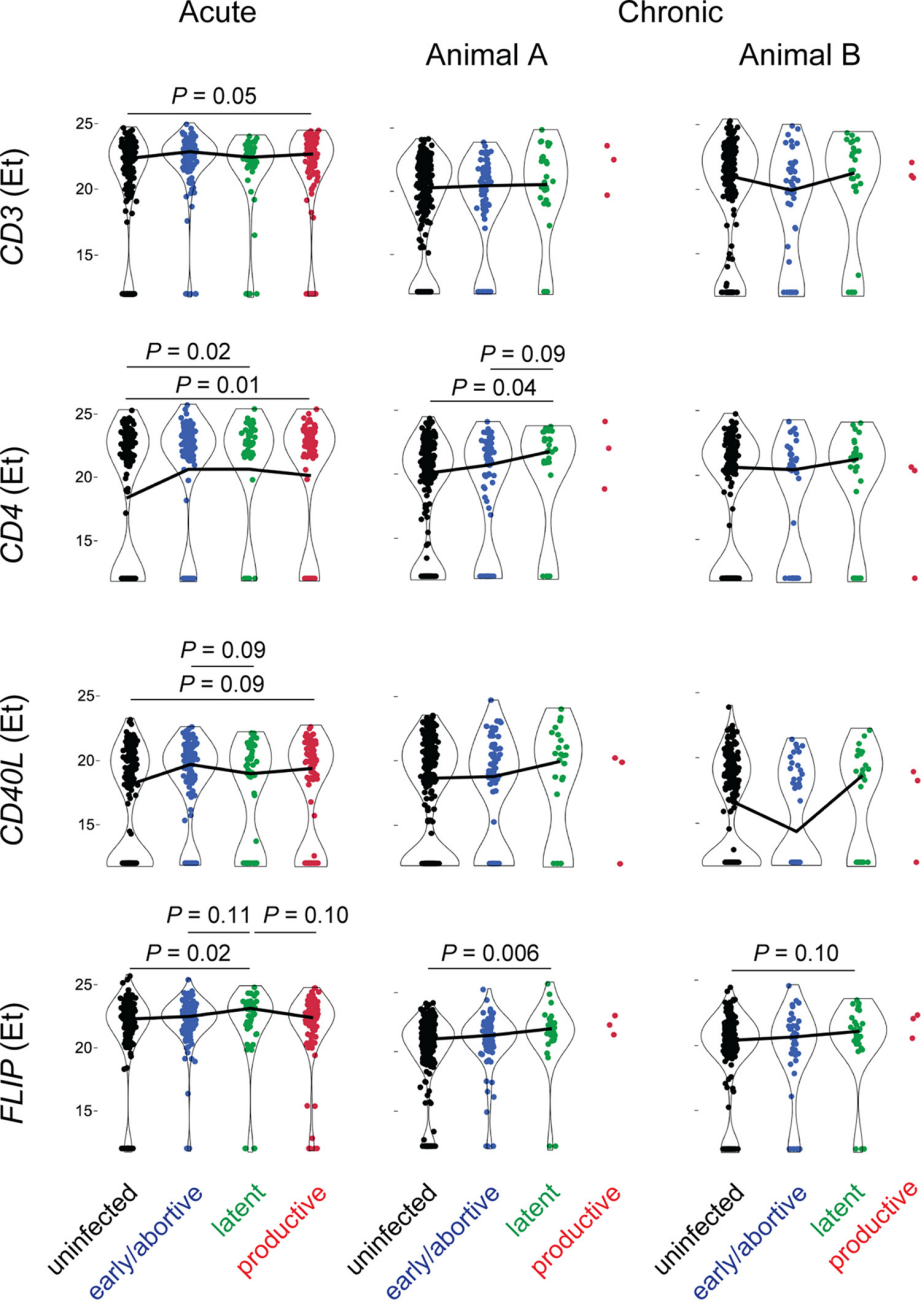
Host genes differentially expressed by SIV-infected CD4 T cells. Expression of host genes was quantified by single-cell RT-qPCR and compared across memory CD4 T cells classified by infection status. Acute and chronic SIV infection macaque lymph node specimens as described above were analyzed. Cell selection criteria were applied as shown in Fig. 4A. Host gene expression is plotted (*E_T_* = *C*_max_ − *Ct*, where *C*_max_ is maximum concentration of drug in serum) for the indicated genes. Lines between violin plots connect mean values. Significant differences between the latently infected cell population and the other cell groups as well as productively infected cells and uninfected cells are indicated (*P*_adj_ < 0.10, Dunn’s test for nonparametric pairwise multiple comparisons following Kruskal-Wallis test, and false-discovery rate adjustment using Benjamini-Hochberg).

To explore host genes differentially expressed by infected cells, we interrogated single CD4^+^ T cells for expression of host genes by RT-qPCR using the same cells isolated from the three untreated acute and chronic SIV-infected lymph node specimens. Again, the cART-suppressed animal was not included in this analysis due to the mixture of uninfected cells with each infected cell. In this proof-of-principle analysis, we selected four host genes, *CD3*, *CD4*, *CD40L*, and *FLIP*, for their high expression positivity rate among memory CD4 T cells by single-cell RT-qPCR (47, 48) and to maximize statistical power. *CD40L* is a marker of T cell activation, while *FLIP* is a prosurvival factor that inhibits both apoptotic and necrotic cell death pathways (65, 66). In productively infected cells, expression of *CD3*, *CD4*, and *CD40L* increased by ~1.3-, 2-, and 2.5-fold, respectively, compared to that in uninfected cells (Fig. 6). Of note, *CD3* and *CD4* transcript levels were elevated in the context of decreased expression of these surface proteins (Fig. 4), consistent with posttranslational mechanisms of downmodulation by viral proteins. Early/abortively infected cells from acute infection also trended toward increased expression of these genes, supporting greater activation of these cells relative to that of uninfected cells. Among latently infected cells, *FLIP* was upregulated consistently across all three acute and chronic infection samples relative to uninfected cells (1.6- to 1.9-fold). Latent cell expression of *FLIP* also trended greater than that in productively infected and early/abortively infected cells in the acute infection setting (*P_adj_* = 0.10 and *P_adj_* = 0.11, respectively). Such host factors may increase the resistance of latent cells to homeostatic cell death processes. Neither *CD3* nor *CD40L* differed between latent and uninfected cells, while *CD4* was elevated 1.8- and 6.5-fold in the acute infection samples and in one chronic infection sample, respectively. The lack of differential *CD3* gene expression suggests that increased surface CD3 protein expression by latently infected cells occurs via a posttranscriptional mechanism. These data demonstrate feasibility of single-cell host gene expression profiling of latently infected cells directly *ex vivo*, including identification of host factors that may play a role in promoting their persistence.

## DISCUSSION

Understanding unique features of latently infected cells will inform strategies to eliminate or modulate HIV-1 reservoirs. Here, we developed a novel single-cell approach to identify and characterize primary cells latently infected *in vivo* by combining flow cytometry and multiplexed RT-qPCR. Unlike many latency models, this approach includes no cellular manipulations or modified viral strains but, rather, captures infected cells directly *ex vivo*. We identified three classes of infected memory CD4 T cells in macaque lymph nodes distinguished by unique combinations of viral RNA and DNA species: (i) replication-active (ipDNA^+^, spliced vRNA^+^), (ii) early or abortive (ipDNA^−^, spliced vRNA^−^, genomic vRNA^+^), and (iii) latent (ipDNA^+^, spliced vRNA^−^, genomic vRNA^+/−^). Integration site sequencing in three latently and two productively infected cells confirmed provirus insertion in the host genome. The ratio of latent to productive cellular infection increased from acute to chronic SIV infection and from chronic to cART-suppressed infection. In proof-of-principle experiments, we demonstrate feasibility of infected single-cell characterization with respect to host protein and gene expression in acute and chronic infection settings, when infected cells are relatively abundant and well-powered comparisons between cell populations are possible.

As expected, infected cells were most frequent during acute infection, when ~40% of all memory CD4 T cells fell into one of the three infection classes. In untreated chronic infection, the frequency was ~1 to 2 logs lower and further reduced by ~1 to 2 logs in cART-suppressed infection. Previous studies of cells harboring integrated SIV DNA in peripheral blood mononuclear cells (PBMC) from acute and chronic SIV infection using qPCR assays on bulk cell populations reported similar infected cell frequencies (54, 55), indicating that the infected cell burden detected by our single-cell method is within the expected range. HIV-1 DNA levels in PBMC of acute, chronic, and suppressed HIV-1 infection tend to be lower, generally ~10^2^ to 10^3^ copies per 10^6^ PBMC, or at most 1% of memory CD4 T cells in acute and chronic untreated infection, and 1 to 10^−1^ copies per 10^6^ PBMC in cART-treated patients (67, 68). These differences may reflect differences in pathogenesis between human HIV-1 infection and SIV infection in rhesus macaques and could limit feasibility of applying the method to HIV-1 specimens.

A surprising finding was the relatively high frequency of latently infected cells during acute infection, at ~8% of memory CD4 T cells, more than half that of productively infected cells (13%). The ~2:1 ratio of productive/latent infection is striking and suggests that a large pool of latently infected cells may be established very early in acute infection. A large proportion of latently infected cells *in vivo* is consistent with multiple blocks in HIV-1 transcription limiting mRNA expression, as observed in patient memory CD4 T cells (69, 70). However, it is also possible that cells transitioning between an early postintegration stage and initiation of proviral transcription share the viral nucleic acid profile used here to define latent infection, resulting in an overestimation of latent cells by this method. Untreated acute infection might be particularly prone to this issue due to massive viral replication in memory CD4 T cells during this window (71). To limit this likelihood, our method incorporates several criteria to maximize specificity for latently infected cells and limit false positives. First, we defined latent infection as cells with *Alu*-LTR signal substantially above the background obtained in the absence of *Alu* primers, reducing the likelihood of including cells harboring unintegrated viral DNA. Second, cells with high genomic vRNA content (in the absence of *tat/rev* mRNA) were excluded to eliminate infected cells with any evidence of viral transcription. Establishment of a substantial pool of latently infected cells during early acute infection is supported by near universal viral rebound following treatment interruption in clinical and preclinical studies when cART is initiated within days of infection (3, 7). Investigation in additional specimens is warranted to corroborate the size of the latent pool during acute infection.

While the proportion of infected cells with intact provirus during untreated acute infection was high, as expected and consistent with early acute HIV-1 infection (72), our estimate of the fraction of latently infected cells harboring intact provirus during cART-suppressed SIV infection was lower than that reported previously, at 16%. In prior analyses, animals started on cART during the first month of infection were found to contain >80% intact proviruses after 9 to 10 months of cART, with lower frequencies observed when cART was initiated after at least a year of infection at 28% and 47% (53, 73). It is not clear why intact proviruses were less frequent in our study, in which cART was initiated 8 weeks postinfection, but we believe that methodologic differences are the most likely explanation. First, our analysis was confined to a subset of infected CD4 T cells defined as latently infected by the presence of integrated DNA and lacking viral transcription. Unlike prior studies interrogating total PBMC or CD4 T cells, our analysis may exclude CD4 T cell subsets or other cells enriched for intact genomes. Unintegrated DNA, phagocytosed DNA, and nonlatent cells transcribing integrated provirus, for example, are not represented in our analysis. Second, the efficiency of the single-cell real-time PCR method used here may vary from that of the PCR and near-full-length genome analyses used elsewhere, possibly failing to detect genomes with nucleotide substitution mutations, resulting in false-negative *gag* or RRE results. It is noteworthy that an analysis of HIV-1 proviruses during untreated Fiebig stage V infection (~30 to 100 days postinfection, similar to when cART was initiated in the cART-treated animal studied here) found only 15 to 30% intact genomes (72). While these results were based on a relatively small sample size, they raise the possibility of deleted genome accumulation after the first month of infection. Such genomes may have persisted in our analysis following a relatively short period of cART. It is also possible that lymph node specimens represent a distinct reservoir relative to that of peripheral blood, though analyses comparing HIV proviruses in matched lymph node and peripheral blood samples have found similar proportions of intact provirus between compartments (74, 75), so this seems less likely.

Latently infected and uninfected cells were similar with respect to expression of most host genes and surface proteins studied, though some novel markers showed differential expression. Increased expression of *FLIP*, a negative regulator of many forms of cell death (65, 66), among latently infected cells was observed consistently in acute and chronic infection. We previously reported *FLIP* upregulation among *in vivo* SIV-infected CD4^+^ T cells lacking spliced viral RNA (48), which we now corroborate for the ipDNA^+^ subset of this population. Elevated *FLIP* may contribute to the persistence of latently infected memory CD4 T cells *in vivo* by augmenting their survival potential, as has been suggested for other prosurvival factors enriched in HIV-1 infected cells such as BIRC5 and Bcl-2 (76, 77). Of note, increased *FLIP* expression among latently infected cells present during acute infection suggests that enrichment of prosurvival factors may be established early in the course of cellular infection rather than selected for over a prolonged period through viral or immune-mediated clearance mechanisms. Interestingly, interfering with FLIP activity using Tat-vFLIP-α2 peptides induces selective death of latently HIV-1-infected CD4^+^ T cells (78), supporting a role for FLIP in infected cell survival. CD3 protein, but not mRNA, expression was also consistently increased among latent cells, which may increase capacity to respond to T-cell receptor (TCR) stimulation and promote clonal expansion (79–81). Latently infected cells were not enriched for IFITM1 or exhaustion markers TIGIT and PD-1, all previously implicated in HIV-1 latency (30, 41). SIV productively infected cells exhibited expected downregulation of surface CD3 and CD4 and modestly increased expression of several activation markers. Clustering analysis of the combined surface marker expression pattern on infected cells did not reveal a profile that defined latently infected cells. These results suggest that latent cellular infection overall is heterogeneous and generally similar to that of uninfected cells, with some notable exceptions. Future studies with a larger sample size of latently infected cells and expanded gene sets will be useful for extending these findings, including features associated with latent cells harboring intact versus defective provirus.

There are several limitations to this study. First, analysis was limited to four animals, only one of which was cART suppressed. Latently infected cells identified during untreated infection may not exhibit the same properties as cells that contribute to persistent viral reservoirs during suppressed infection. In addition, due to the rarity of latently infected cells during suppressed infection, we were able to establish only their viral nucleic acid profile and not their phenotype by single-cell surface or host gene expression profiling. Increasing the assay throughput to interrogate >10^5^ cells with single-cell resolution will be required to address these gaps.

Recent advances in high-throughput and high-content single-cell technologies have revolutionized investigations of biological differences between cells, including in the context of HIV-1 infection (41, 42, 82). Our novel single-cell method offers a valuable strategy for assessing the basic biologic properties that distinguish latently infected cells as they exist *in vivo*, with several advantages over existing *in vitro* and *ex vivo* approaches. First, by analyzing individual cells, we avoid ambiguity regarding interpretation of signals averaged across populations of bulk cells. Second, cells are not manipulated following isolation, with no *ex vivo* culture or stimulation, preserving transcriptional profiles more reflective of their *in vivo* state. Third, by combing ipDNA PCR with the high-dimensional analyses of multiplexed RT-qPCR and polychromatic flow cytometry, up to ~150 parameters can be measured for each cell simultaneously in relatively high throughput (e.g., ~100 gene targets by multiplex qPCR; ~50 surface markers using current state-of-the-art flow cytometers). The approach may be particularly useful for validating single-cell transcriptome sequencing (RNA-seq) results with benefits including increased power due to fewer analytes and greater sensitivity for low-copy genes. Of note, this approach is readily adapted to the study of HIV-1-infected cells, as the assays target regions present in both SIV and HIV-1.

## MATERIALS AND METHODS

### Animals and SIV infection.

Four colony-bred Indian-origin male rhesus macaques of ages 3 to 8 years were infected with SIVmac251 administered either intravenously (50% median infective dose [MID_50_] of 100; VRC animal protocol 356, animal AY69) or intrarectally (50% tissue culture infective dose [TCID_50_] of 500; Bioqual animal protocol 15-088, animals BS91, T594, and T595). Virus preparations of 1.0 mL were inoculated via the saphenous vein or a lubricated feeding catheter inserted into the rectum. Infection duration was 10 days (AY69, viral load 24,159,248 RNA copies/mL), 15 weeks (T595, <50 copies/mL), or 7.5 months (T594 and BS91; 33,600 and 175,538 copies/mL, respectively). T595 SIV viral load peaked at 77,156,000 17 days postinfection and fell to 17,450 at week 8, when daily subcutaneous cART consisting of tenofovir disoproxil fumarate (TDF), emtricitabine (FTC), and dolutegravir (DTG) was initiated. Viral load was suppressed to 300 at week 9 and sustained below the assay limit of detection (<50 copies/mL) weeks 10 through 15.

### Flow cytometric cell phenotyping and sorting.

Cryopreserved mesenteric lymph nodes were surface stained for memory CD4 T cell phenotyping and isolation by flow cytometry. Surface protein expression for each cell was assessed by staining with the following fluorescent monoclonal antibodies (BD Biosciences, unless noted otherwise): CD4-BV786 (clone L200, lot 8099675), CD8-BUV496 (clone RPA-T8, lot 7193592), CD3-BV650 (clone SP34-2, lot 7018763), CD95-BUV737 (clone DX2, lot 8072740), CD28-BV711 (clone CD28.2, lot 236771), CD14-BV510 (Biolegend, clone M5E2, lot 237626), CD16-BV510 (Biolegend, clone 3G8, lot 238638), CD20-BV510 (Biolegend, clone 2H7, lot 244953), CXCR3-PE-Cy5 (clone 1C6, lot 7040790), CD38-PE (NHP reagent program, clone OKT10, lot 060816CK), CD69-BUV395 (clone FN50, lot 7108931), ICOS-Alex Fluor 700 (Biolegend, clone C398.4A, lot 227702), HLA-DR-APC-H7 (clone L243, lot 9051562), HLA-ABC-BV605 (Biolegend, clone W6/32, lot 256257), TIGIT-PerCP-eFluor 710 (ThermoFisher/eBioscience, clone MBSA43, lot 4290851), CXCR5-FITC (ThermoFisher/eBioscience, clone MU5UBEE, lot 4325074), PD-1-PE-eFluor 610 (ThermoFisher/eBioscience, clone eBioJ105, lot 4323887), integrin β7-BV421 (clone FIB 504, lot 6270954), CCR6-PE-Cy7 (clone 11A9, lot 8152573), IFITM1 (abcam, rabbit polyclonal, lot GR110366-1), anti-rabbit allophycocyanin (APC; Jackson Immuno Research, lot 126640). Cells were first stained with aqua live/dead stain (ThermoFisher/Invitrogen, lot 1596087) and anti-IFITM1 antibody, then washed and stained with a cocktail of all the remaining antibodies, including secondary APC-labeled antibody for IFITM1. For assay validation experiments, 3D8 cells (NIH HIV Reagent Program, Division of AIDS, NIAID, NIH: ARP-13239, contributed by Mario Roederer and Joseph Mattapallil) were sorted by flow cytometry at limiting dilutions in replicate. Cell sorting was performed immediately after specimen thawing and staining without any *in vitro* manipulation or culture. For lymph node specimens from the untreated macaques, 1,000 to 2,000 memory CD4 T cells were isolated and deposited into 96-well PCR plates at one cell per well. For the cART-suppressed animal, replicates of 10 to 300 cell dilutions were collected and the dilution containing a single infected cell was identified by Poisson distribution probability estimation based on viral nucleic acid positivity of <33%. Flow cytometry data analysis was performed using FlowJo software (BD Biosciences), v. 9 and 10. *t-*distributed stochastic neighbor embedding (tSNE) was used to visualize population representation and clustering results with the recommended parameters (perplexity of 30 and iteration of 1,000) in FlowJo v. 10 (83). Markers used in the gating strategy were not included in generating the tSNE plots.

### cDNA synthesis, nuclear lysis, and quantitative PCR.

Cells were sorted directly into 10 μL RT-PCR buffer in 96-well PCR plates for immediate lysis and gene-specific multiplexed primer-based reverse transcription for 15 min followed by 3 cycles of preamplification using SuperScript III Platinum one-step qRT-PCR kit (ThermoFisher/Invitrogen). mRNA primers (45 nM) were as follows: SIV *gag* and *tat/rev* (48) and TaqMan assays (ThermoFisher/Invitrogen) for *CD3E* (Rh01062242_m1), *CD4* (Rh02621720_m1), *FLIP* (Hs01116280_m1), and *CD40LG* (Rh02787995_m1). The nuclear membrane was then lysed by addition of 5 μL of 0.3 mg/mL proteinase K (Roche; diluted in water immediately before use) to the cDNA mixture, incubation for 3 h at 55°C, and proteinase K inactivation for 10 min at 95°C. Five microliters of Platinum *Taq* reaction (ThermoFisher/Invitrogen) was added to the preamplified cDNA and nuclear genomic DNA mixture for further preamplification by PCR for 15 cycles, using the residual RT primers remaining in the reaction and with the addition of primers for *Alu*(lambda)-LTR (*Alu* forward: 5′-TCCCAGCTACTCGGGAGGCTGAGG, *Alu* reverse: 5′-CTCCCAAAGTGCTGGGATTACAGG, Lambda/LTR: 5′-CCTAGCCACGTAAGCGAAACTAGGGACTAATTTCCATAGCCAGCCAAAT) at 180 nM and 45 nm for *Alu* and Lambda/LTR primers, respectively. The resulting preamplified cDNA and genomic DNA were diluted 5-fold in DNA suspension buffer (Teknova) and quantified by qPCR performed for 45 cycles on a QuantStudio 6 (ThermoFisher/Applied Biosytems) using Platinum *Taq* (ThermoFisher/Invitrogen) and primers and probes specific for SIVmac251 *gag*, *tat/rev* (48), *RRE* (forward: 5′-GGTGGCACCTCAAGAAATAA, reverse: GCACTATCCCAGCCAATAAA, probe: 5′-/56-FAM/AACCCAAGA/ZEN/ACCCTAGCACAAAGACC/3IABkFQ/), lambda-LTR (lambda: 5′-CCTAGCCACGTAAGCGAAACTAG, LTR forward: 5′-CTTAGAAAAGGAAGAAGGCATCATACCA, probe: 5′-/56-FAM/CAGGATTAC/ZEN/ACCTCAGGACCAGGAATTAGATACCC/3IABkFQ/), and the TaqMan assays for *CD3*, *CD4*, *CD40LG*, and *FLIP* described above. The RRE assay was absent during RT and preamplification as amplicons encompassing RRE generated from unspliced viral RNA, and viral DNA by *tat/rev* primers served as the template for the RRE qPCR. qPCR data were converted to *E_T_* values (40 − *Ct*) to reflect relative transcript levels. Amplification efficiency was calculated as E = −1 + 10^(−1/slope)^. Absolute RNA copies were calculated as follows: copy number = 2^(*E_T_* − *E_T_*^minimal^), where minimal *E_T_* is 13 for *tat/rev* and *gag*, based on the previously determined average value for a single RNA copy by this method (48, 49), and minimal *E_T_* is 10 for RRE (RNA or DNA), based on the distribution of observed *E_T_* values. For host gene expression, *E_T_* values of <13 were pinned at 12 to reflect less than one copy (49).

### Sequencing across integration sites.

Integrated viral genomic DNA from FACS-sorted single cells preamplified for 15 cycles was further PCR amplified for an additional 45 to 90 cycles using lambda and *Alu* primers. The resulting PCR products were separated on 2% agarose gel supplemented with SYBR safe DNA gel stain (Invitrogen), and all visible bands were excised manually. DNA was extracted using MinElute gel extraction kit (Qiagen) and sequenced by Sanger method using the Lambda primer (ACGT, Inc.). Unambiguous sequencing reads were aligned with SIVmac239 sequence using Geneious (Biomatters, Inc.) and with M. mulatta using the BLAST/BLAT tool in Ensembl Genome Browser.

### Statistical analysis.

Differential protein and gene expression between groups of infected cells was determined using Dunn’s test for nonparametric pairwise multiple comparisons following Kruskal-Wallis test and false-discovery rate adjustment using Benjamini-Hochberg (*P*_adj_ < 0.10).

### Ethics statement.

Research was conducted under approved animal use protocols in an Association for Assessment and Accreditation of Laboratory Animal Care accredited facility in compliance with the Animal Welfare Act and other federal statutes and regulations relating to animals and experiments involving animals and adheres to principles stated in the Guide for the Care and Use of Laboratory Animals, NRC Publication, 2011 edition. Animal protocols and procedures were reviewed and approved by either the NIH Vaccine Research Center IACUC (VRC protocol 356) or the Animal Care and Use Committee of both the US Army Medical Research and Material Command (USAMRMC, protocol 11355007.07) Animal Care and Use Review Office and the Institutional Animal Care and Use Committee of Bioqual, Inc. (protocol number 15-088), where nonhuman primates were housed for the duration of the study. Bioqual, Inc. and the USAMRMC are in full compliance with the Animal Welfare Act and Public Health Service Policy on Humane Care and Use of Laboratory Animals.

### Data availability.

The data sets that support the findings of this study are available from the corresponding author upon reasonable request.
